# Supplementary material for: Single-cell profiling of penta- and tetradactyl mouse limb buds identifies mesenchymal progenitors controlling digit numbers and identities
Source: Nat Commun. 2025 Jan 31;16:1226. doi: 10.1038/s41467-025-56221-1 (PMC11785988; doi:10.1038/s41467-025-56221-1)
Supplement: Supplementary file 1 — Supplementary Information [file 41467_2025_56221_MOESM1_ESM.pdf]

Supplementary information

Supplementary Figure 1

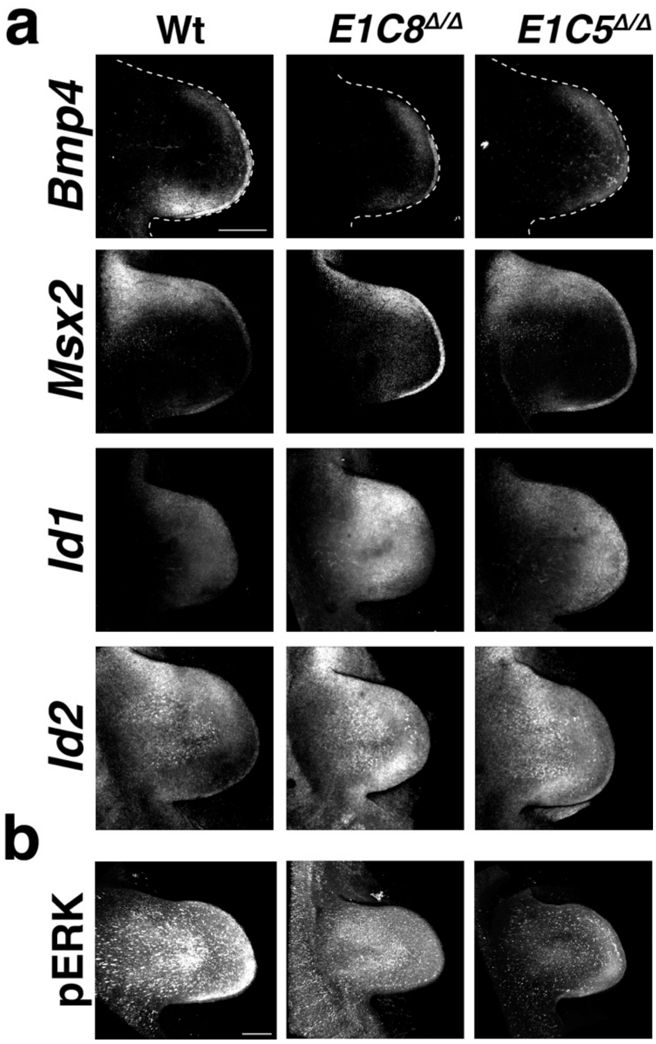

Supplementary Figure 1. Alterations in BMP and FGF pathway activities. Comparative RNA-FISH analysis of wildtype and mutant forelimb buds at E10.75 (37-39 somites). Greyscale are shown for all analysis. **a** Analysis of BMP4 ligand in the feedback signaling system and the downstream BMP activity sensors *Msx2*, *Id1* and *Id2*. Scale bar: 300 µm. (n=3 replicates per genotype) **b** Whole mount immunofluorescence analysis of phospho-ERK (pERK) activity in E10.75 forelimb buds (n=2 replicates per genotype). Scale bar: 200µm. All limb buds are oriented with anterior to the top and posterior to the bottom.

Supplementary Figure 2

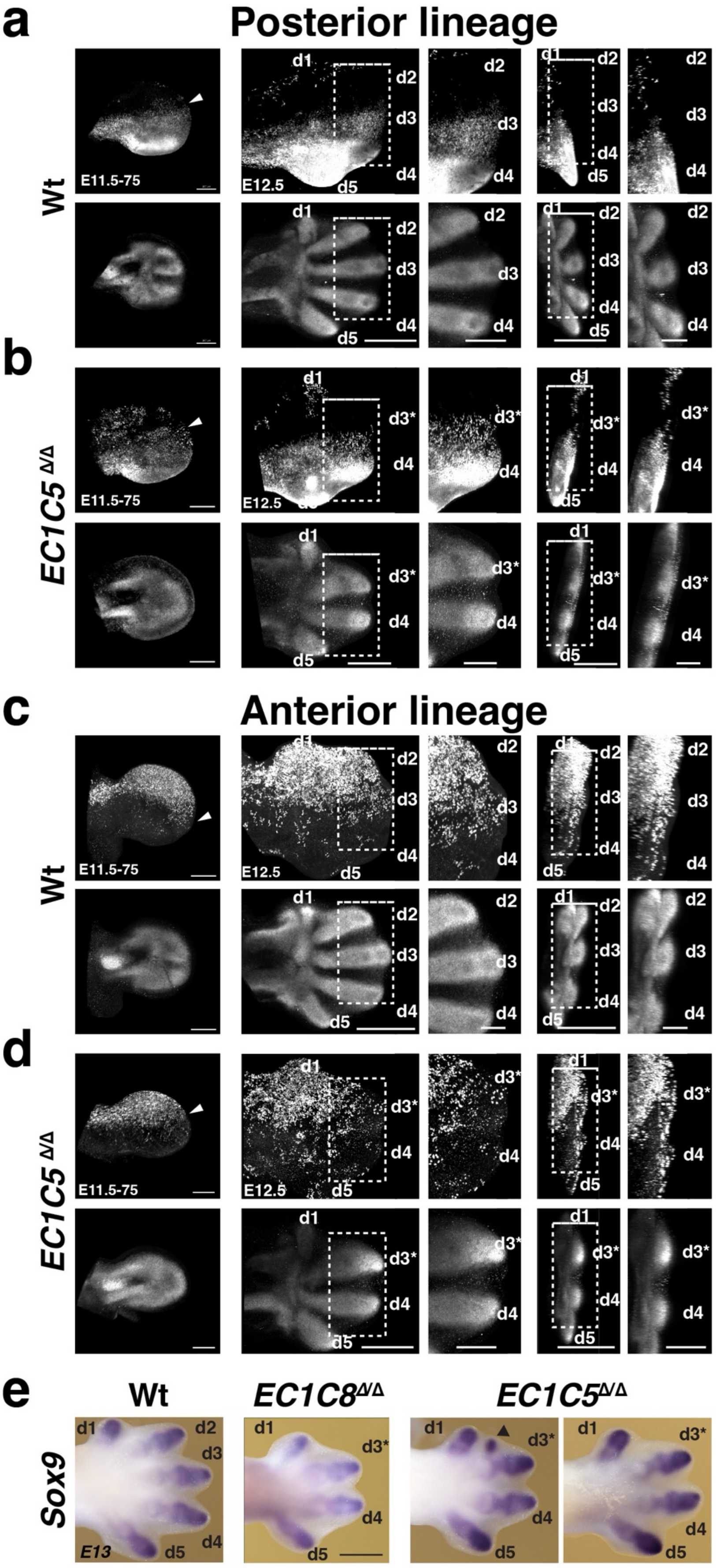

Supplementary Figure 2. Greyscale for the lineage analysis shown Figure 2 and detection of *Sox9* expression in the most anterior interdigit of some *E1C5*$^{\Delta/\Delta}$ forelimb buds. The greyscale images for all posterior and anterior lineage analysis in forelimb buds shown in Figure 2 are included here. **a, b** Posterior lineage in wildtype and tetradactyl *E1C5*$^{\Delta/\Delta}$ forelimb bud forelimb buds show the posterior lineage (upper panels) and *Sox9* domain in the developing cartilage primordia (lower panels). Arrowheads indicate the anterior boundary of the posterior lineage. **c, d** Anterior lineage analysis in the wild-type and *E1C5*$^{\Delta/\Delta}$ tetradactyl limb buds. Arrowheads indicate the posterior boundary of the anterior lineage. Scale bars for panels a-d: left panels: 200µm; right panels: 500 µm for overviews, 250 µm for insets. **e** Conventional whole mount RNA *in situ* hybridization (Malkmus et al., 2021, doi.org/10.1038/s41467-021-25810-1) shows the *Sox9* expression pattern in wildtype and *E1C8*$^{\Delta/\Delta}$ and *E1C5*$^{\Delta/\Delta}$ tetradactyl forelimbs at E13.5. The arrowhead points to the *Sox9*-positive condensation in the interdigit between digit d1 and d3* in a *E1C5*$^{\Delta/\Delta}$ forelimb (n=2/5), while no ectopic *Sox9*-positive condensations were detected in *E1C8*$^{\Delta/\Delta}$ forelimbs (n=0/3). Scale bar: 250µm. All limb buds are oriented with anterior to the top and posterior to the bottom.

Supplementary Figure 3

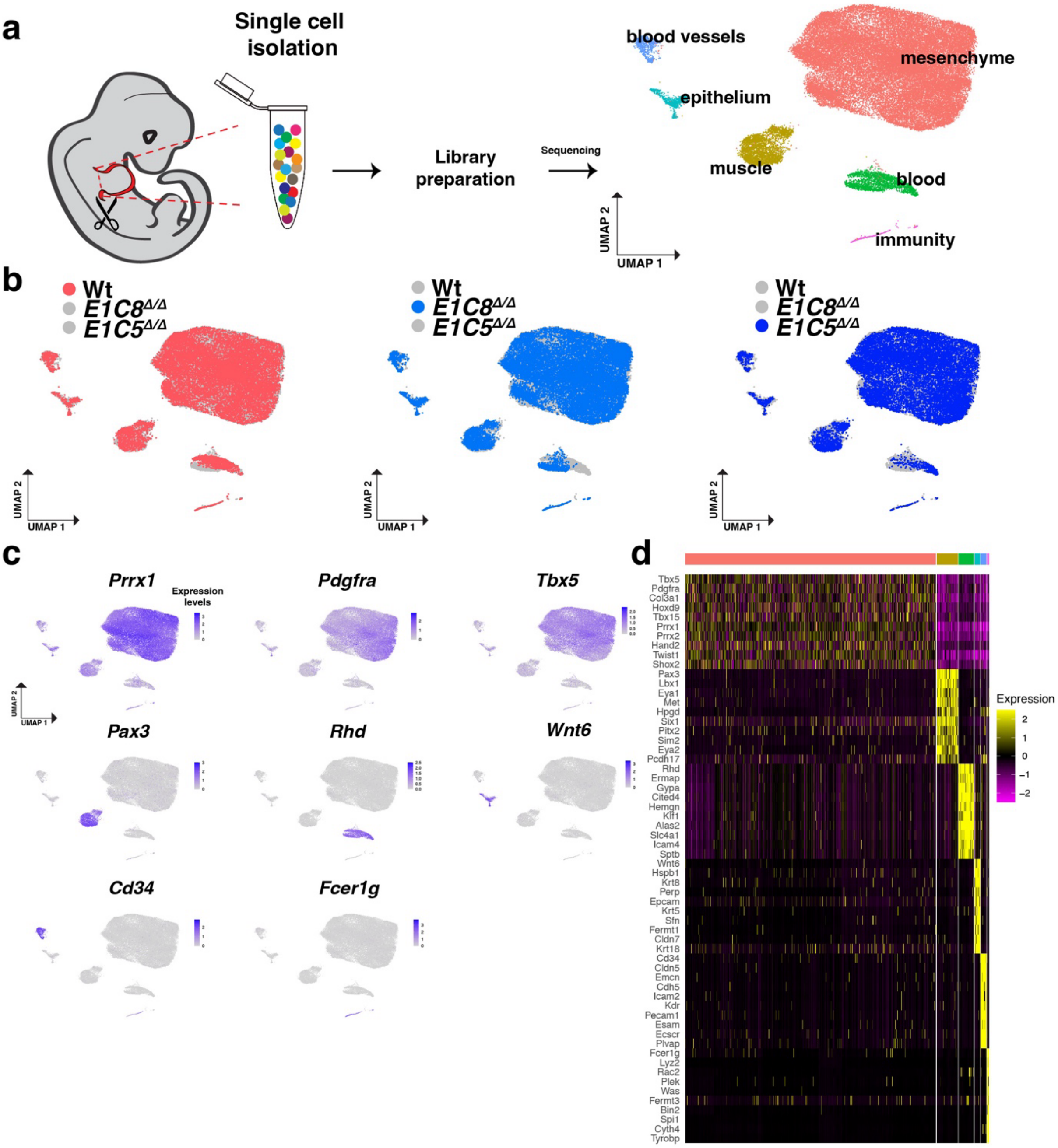

Supplementary Figure 3. Single cell RNA-sequencing pipeline, initial data processing and analysis. a Scheme showing the experimental procedure. Forelimb bud pairs from wildtype, *E1C8*$^{\Delta/\Delta}$ and *E1C5*$^{\Delta/\Delta}$ embryos (n=3 biological replicates per genotype) were dissected and dissociated into single cells, libraries prepared and sequenced. After initial validation and filtering, an initial UMAP clustering of the pooled scRNA-seq datasets from all three genotypes was performed using expression score of genes specific for each of the tissue types detected (right-most panel). **b** Separate

UMAP visualisation of the initial clustering for each genotype. Left panel: wildtype in red, middle panel: *E1C8*$^{\Delta/\Delta}$ in blue, right panel: *E1C5*$^{\Delta/\Delta}$ in darker blue against the other two genotypes (grey). **c** UMAP visualisation of established limb bud mesenchymal markers *Prxx1*, *Pdgfra* and *Tbx5* (top row). In addition, UMAP visualization of key markers of all other cell and tissue types that are not expressed by limb bud mesenchymal cells are shown (middle and bottom row) **d** Global heatmap including the top 10 enriched genes expressed in each of the cluster identified (panel a).

Supplementary Figure 4

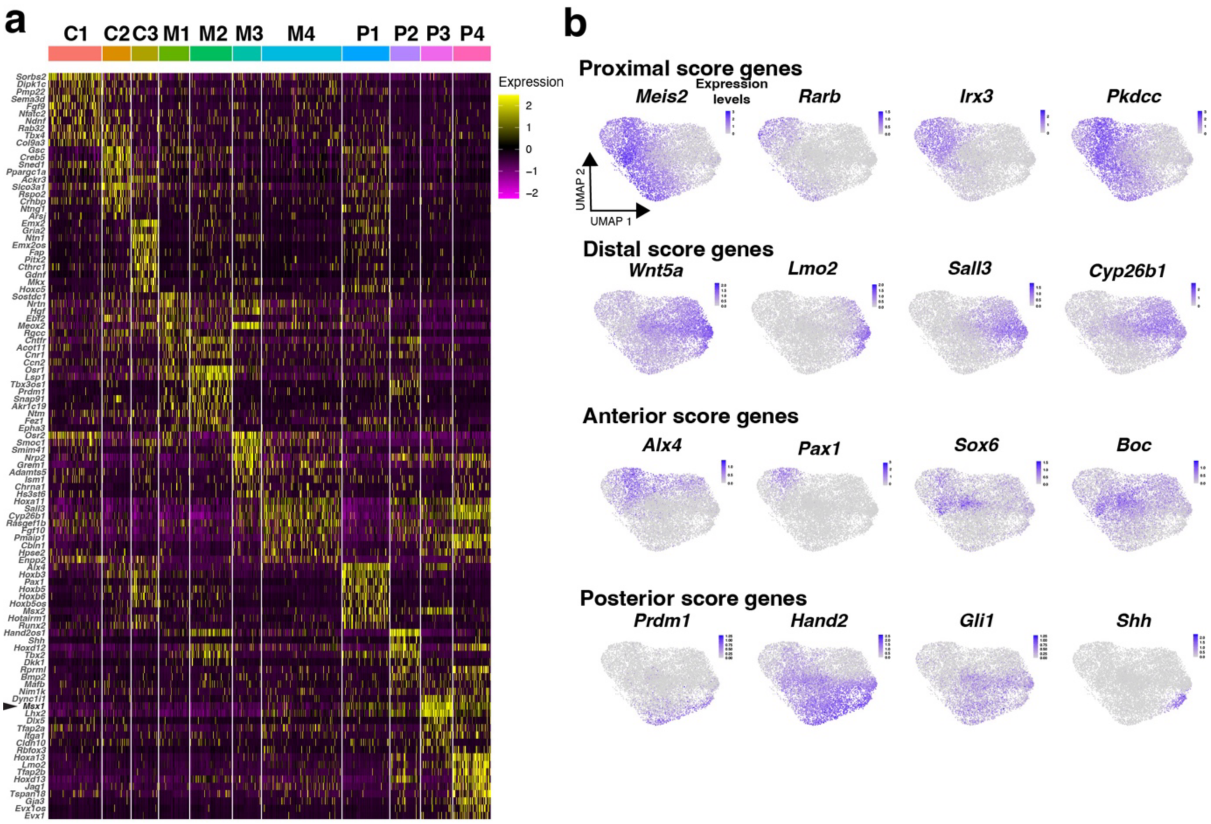

Supplementary Figure 4. Extended signature and spatial scores for the wildtype mesenchymal clusters. a Heatmap generated using top enriched genes irrespective of spatial expression and function from all 11 mesenchymal clusters. Chondrocytes: C1-C3; mesenchyme: M1-M4; *Msx1*+ progenitors: P1-P4. **b** UMAP embedding of genes that were selected to define the scores for the proximal to distal and anterior to posterior limb bud axes in Figure 3c. Proximal: *Meis2*, *Rarb*, *Irx3*, *Pkdcc*; distal: *Wnt5a*, *Lmo2*, *Sall3*, *Cyb26b1*; anterior: *Alx4*, *Pax1*, *Sox6*, *Boc*; posterior: *Prdm1*, *Hand2*, *Gli1*, *Shh*. For all UMAPs, cells were plotted in random order: FeaturePlot(object, order=F).

Supplementary Figure 5

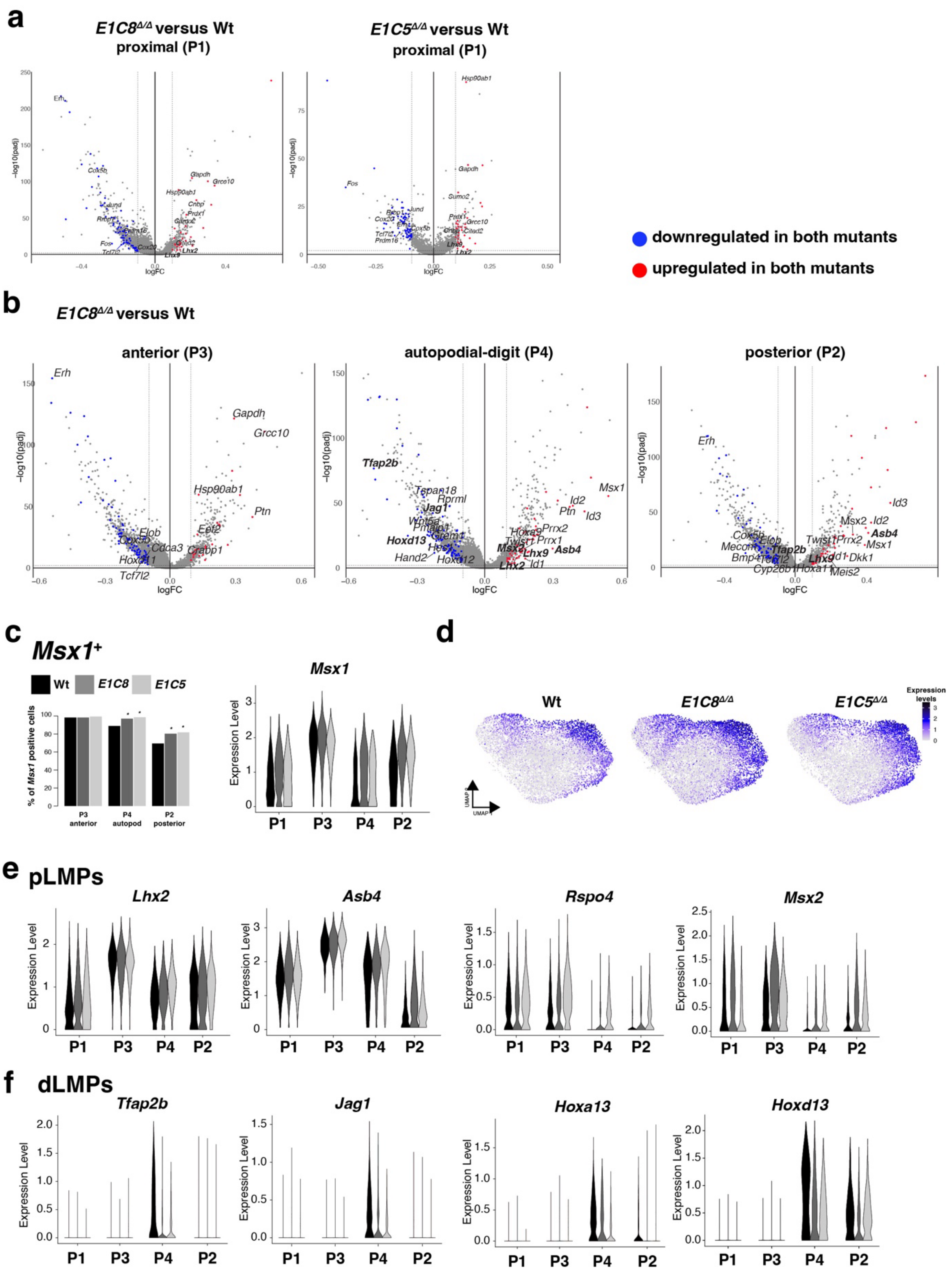

Supplementary Figure 5. Differential gene expression in *Msx1*+ progenitor clusters and selection of pLMP and pLMP score signature genes. a Volcano plots

showing the differentially expressed genes (DEGs) in the proximal cluster (P1) by comparing wildtype to *E1C8*$^{\Delta/\Delta}$ and wildtype to *E1C5*$^{\Delta/\Delta}$ limb buds calculated using Wilcoxon Rank-Sum Test. The expression of DEGs indicated in blue is downregulated in Grem1 tetradactyl limb buds in comparison to wildtype limb buds. Conversely, the expression of DEGs in red is upregulated in Grem1 tetradactyl limb buds. Fold changes (x-axis) are shown as log2 values, while significance (y-axis) is shown as log10 of the adjusted p-values. **b** Volcano plots showing the DEGs in the anterior (P3), autopodial-digit (P4) and posterior (P2) progenitor clusters by comparing wildtype to *E1C8*$^{\Delta/\Delta}$ limb buds. The expression of DEGs indicated in blue is downregulated. Conversely, the expression of DEGs indicted in red are upregulated. Fold changes (x-axis) are shown as log2 values, while significance (y-axis) is log10 of the adjusted p-values. **c** Left panels: Bar plots displaying the fraction of *Msx1*+ expressing progenitors (score >0) in all three genotypes in the 3 distal clusters (P2-4) and in the total limb bud mesenchyme (Source data are provided as source data file). Asterisks indicate statistical significance (p-values ≤0.001). Right panels: violin plot analysis showing the relative *Msx1* expression levels in clusters P1- P4. **d** UMAP embedding showing the distribution of *Msx1*+ expressing progenitors in the three genotypes. **e, f** Violin plot shows the relative expression levels of the signature genes used to define the pLMP and dLMP score in the proximal (P1), anterior (P3), autopodial-digit (P4) and posterior clusters (P2). Expression levels for all three genotypes are shown.

Supplementary Figure 6

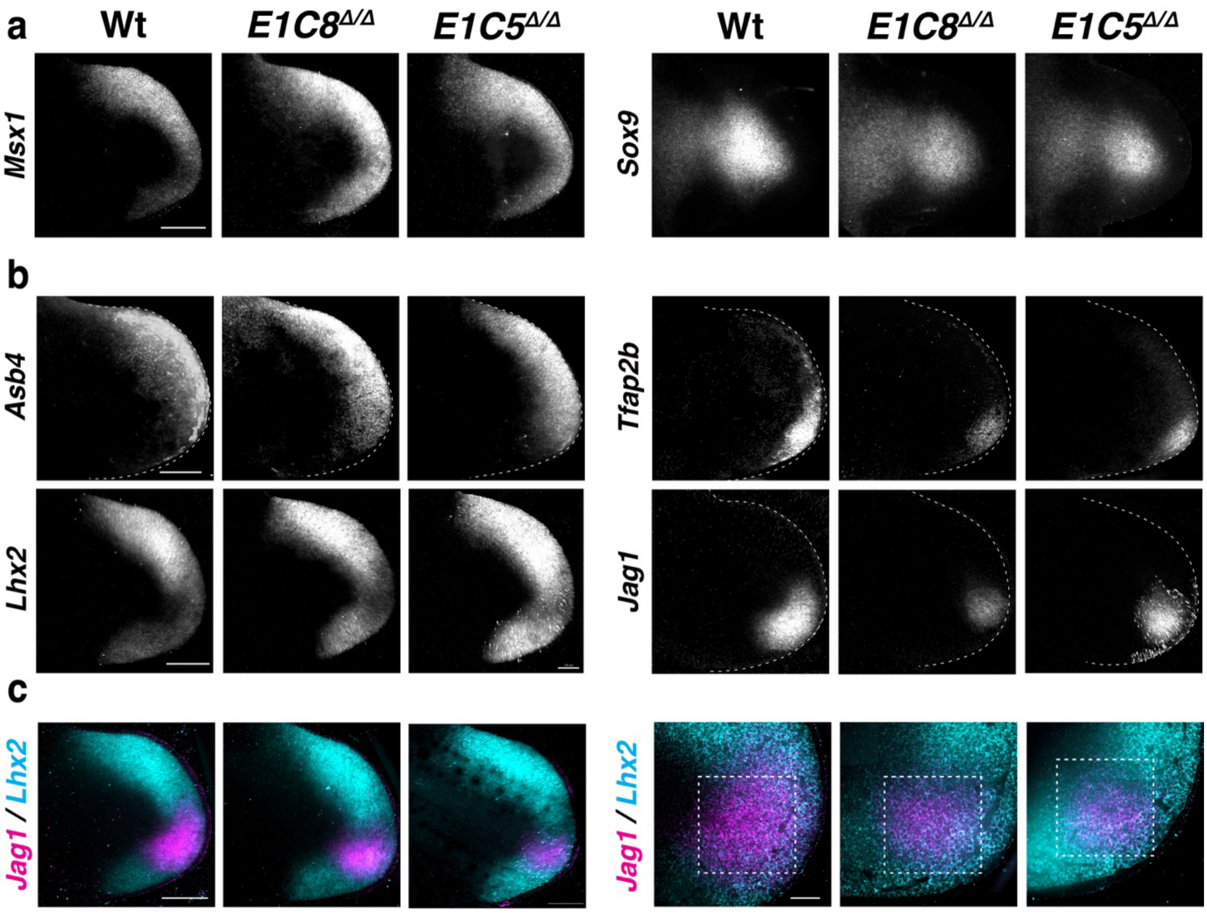

Supplementary Figure 6. Additional RNA-FISH analysis of wild-type and Grem1 tetradactyl forelimb buds. a *Msx1* (left panels) and *Sox9* expression (right panels) for the wild-type, *E1C8*$^{\Delta/\Delta}$ and *E1C5*$^{\Delta/\Delta}$ forelimb buds shown in Fig. 5a. (n=6 *Msx1* and n=3 *Sox9* replicates per genotype). **b** Top panels: *Asb4* and *Tfab2b* expression for the wild-type, *E1C8*$^{\Delta/\Delta}$ and *E1C5*$^{\Delta/\Delta}$ forelimb buds shown in Fig. 5c. Bottom panels: *Lhx2* and *Jag1* expression for the wild-type, *E1C8*$^{\Delta/\Delta}$ and *E1C5*$^{\Delta/\Delta}$ forelimb buds shown in Fig. 5c. (n=3 replicates per genotype). **c** Whole Mount RNA-FISH images showing *Lhx2* (cyan) and *Jag1* (magenta) expression in wildtype and mutant forelimb buds used for the optical sections shown in Fig. 5d (note that these are limb buds different from the ones shown in Fig. 5c). Scalebars: 200µm; enlargements in panel c: 50µm. The white rectangles indicate the enlargements shown in Fig 5d. (n=3 per genotype).

Supplementary Figure 7

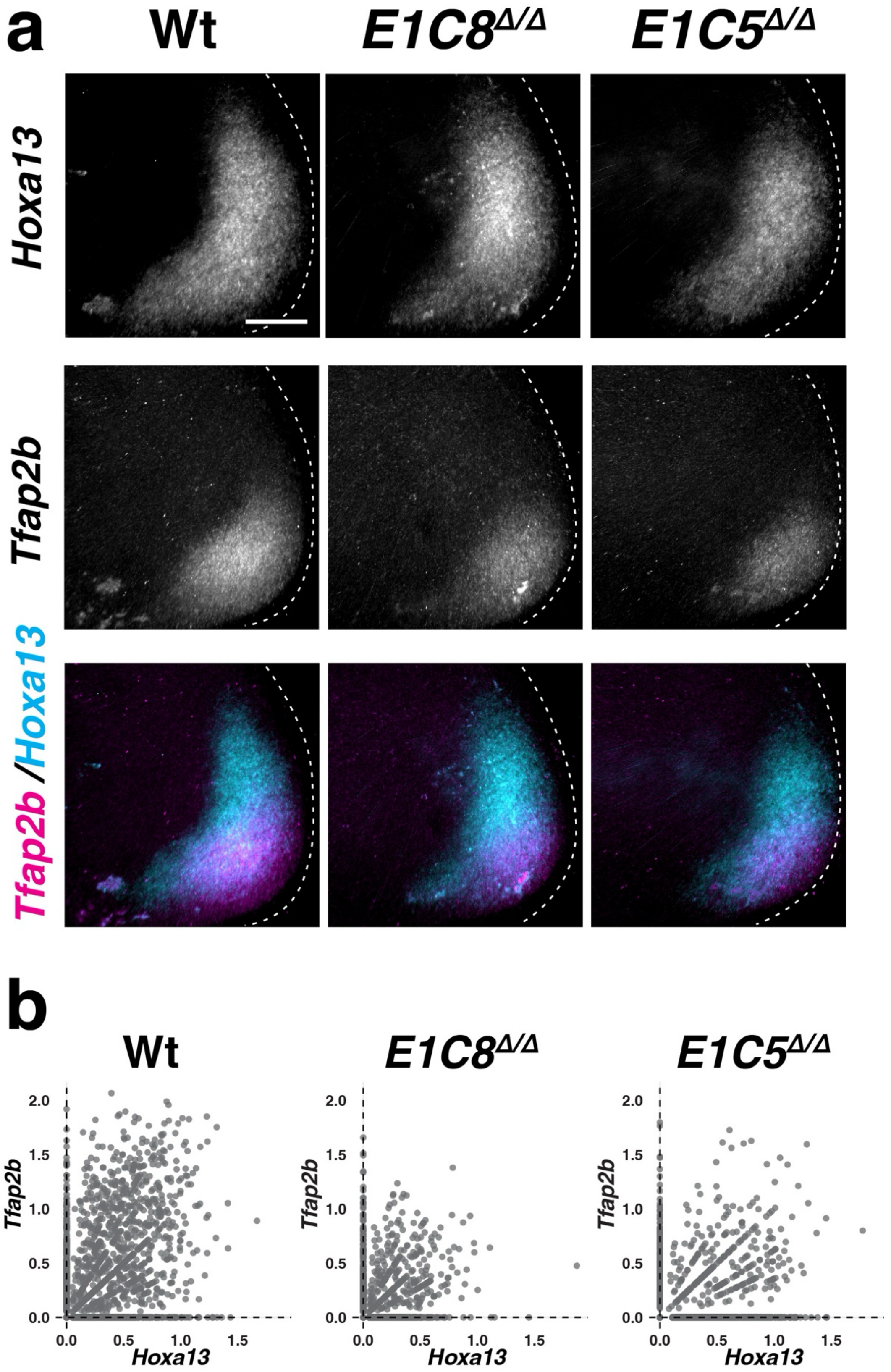

Supplementary Figure 7. Reduction of co-expressing dLMPs in Grem1 tetradactyl limb buds. a The spatial distribution of *Hoxa13* and *Tfap2b* genotypes is

shown individually (grey scale, top and middle panels) and as overlap (bottom panels) in the same forelimb buds. Scalebars: 100µm. (n=3 replicates per genotype). **b** Scatterplot representation of single mesenchymal cells identifies co-expressing *Hoxa13* (x-axis) and *Tfap2b* (y-axis) LMPs in wildtype, $E1C8^{\Delta/\Delta}$ and $E1C5^{\Delta/\Delta}$ forelimb buds. In agreement with RNA-FISH, the number of co-expressing cells is significantly reduced between wildtype and $E1C8^{\Delta/\Delta}$ and wildtype and $E1C5^{\Delta/\Delta}$ forelimb buds (two-sided Fisher test, Source data are provided as source data file).

Supplementary Figure 8

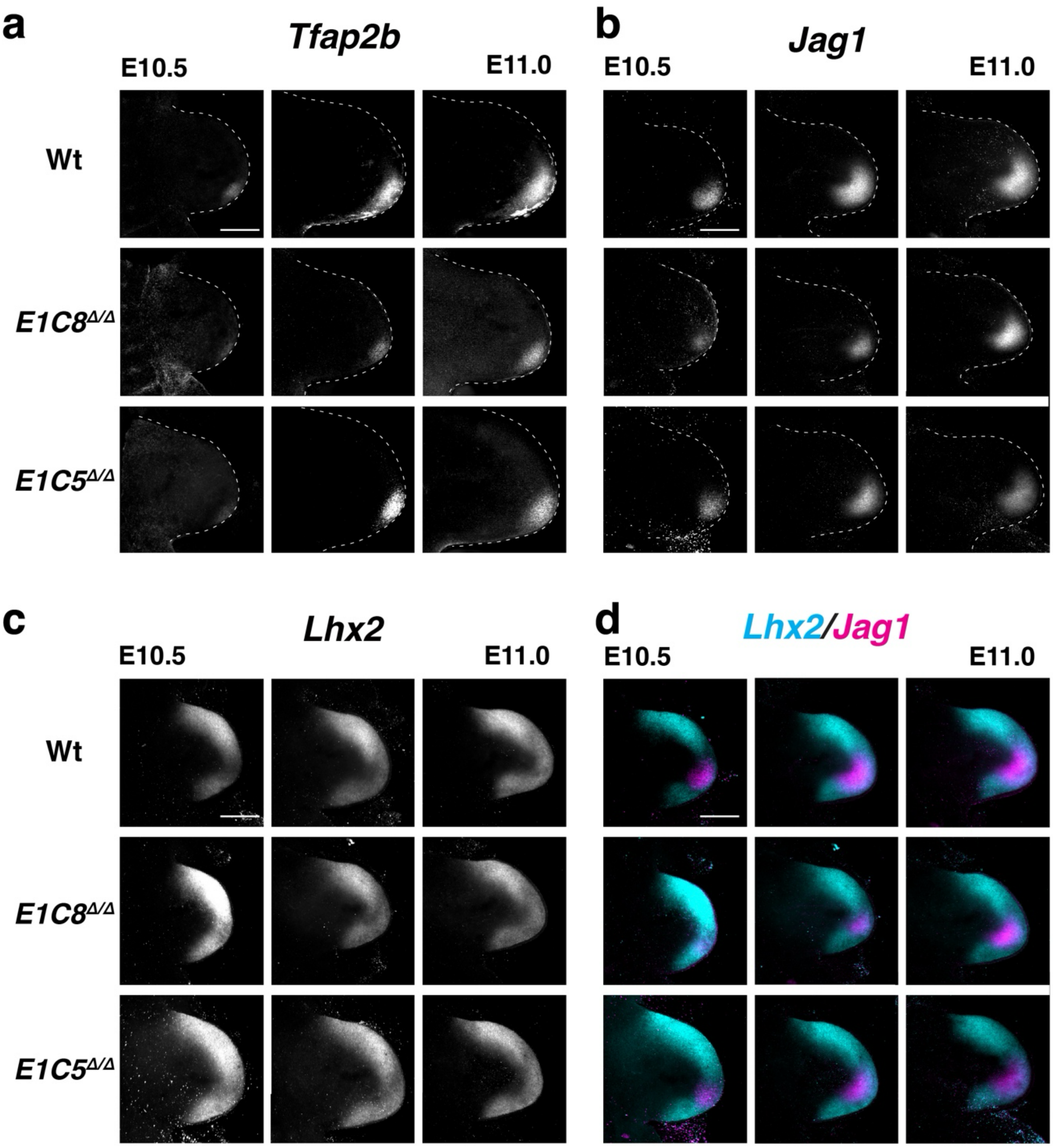

Supplementary Figure 8. Spatio-temporal progression of the dLMP and pLMP domains. a-d Comparative RNA-FISH analysis of a temporally ordered series of wildtype and E1C8$^{\Delta/\Delta}$ and E1C5$^{\Delta/\Delta}$ forelimb buds (E10.5 to E11.25, range: 36-43 somites) illustrates the temporal progression in spatial gene expression (n=3 replicates per genotype). All limb buds are oriented with anterior to the top and posterior to the bottom. Scale bars: 300µm. **d** Merge of the *Jag1* (magenta) and *Lhx2* (cyan) expression patterns.

Supplementary Figure 9

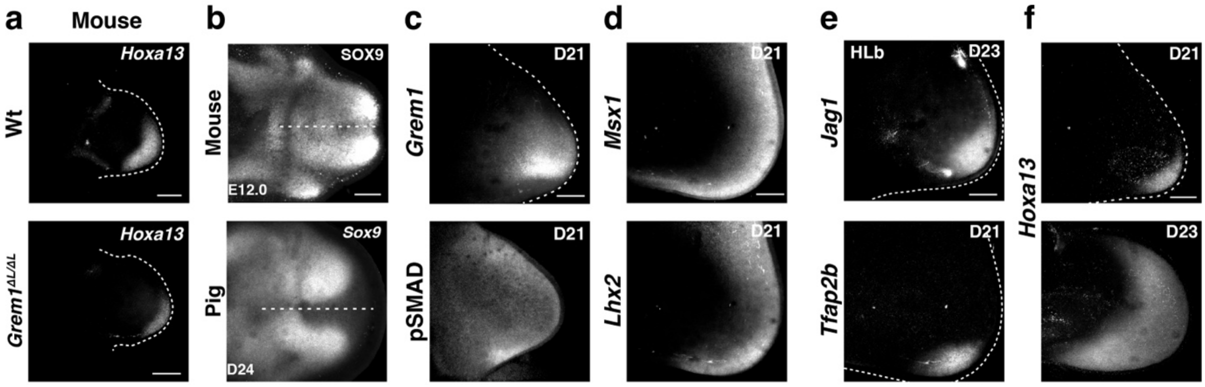

Supplementary Figure 9. *Hoxa13* expression in *Grem1*$^{ΔL/ΔL}$ and analysis of LMP signature genes in developmentally younger pig limb buds. a *Hoxa13* expression in wild-type and *Grem1*-deficient forelimb buds at E10.75. Scale bars: 200µm. (n=3 replicates per genotype). b, Upper panel: SOX9 protein distribution in *E1C5* $^{Δ/Δ}$tetradactyl mouse forelimb buds at E12.5 (n=6). Lower panel: *Sox9* expression in pig forelimb buds at D24 (n=3). c, Spatial distribution of *Grem1* (RNA-FISH) and pSMAD activity (immunofluorescence) in pig forelimb buds at gestational D21. **d** *Msx1* and *Lhx2* expression in pig forelimb buds at D21. **e** *Jag1* (hindlimb bud D23) and *Tfap2b* expression (forelimb bud D21) in pig limb buds. Note: pig hindlimb buds at D23 are equivalent to forelimb buds at D21. **f** The spatial distribution of *Hoxa13* in pig forelimb buds at D21 and D23. All analysis shown in panels c-f n=3 independent biological replicates analyzed. Scale bars panels b-f: 300 µm. All limb buds are oriented with anterior to the top and posterior to the bottom.

Supplementary Table 1. Genotyping primers.
All references are listed in the main text.

Primer sequence	Reference
Mutant allele EC1 Forward: GGGACAAGTCACAGATCTTTTTG	Malkmus et al., 2021
Mutant allele EC1 Reverse: TCCTTCATGTCTCGTTTTGTTTT	Malkmus et al., 2021
Wt allele EC1 Forward: TCCAGTTAAATGCAAAAAGGAAA	Malkmus et al., 2021
Wt allele EC1 Reverse: CTCTTCCTTCATCTCTCCTAGCC	Malkmus et al., 2021
Mutant allele CRM5 Forward: CTCCCATATGCTCACCGGTTT	Malkmus et al., 2021
Mutant allele CRM5 Reverse: GGTGGGAGTGGAGTTTGACC	Malkmus et al., 2021
Wt allele CRM5 Forward: GGTAAGGAGCCAGCCATATTTG	Malkmus et al., 2021
Wt allele CRM5 Reverse: GGTGGGAGTGGAGTTTGACC	Malkmus et al., 2021
Mutant allele CRM8 Forward: CCATTCCTAAAACCCAAGCA	This study
Mutant allele CRM8 Reverse: TGCACTTGGTAAGCATTGGA	This study
Wt allele CRM8 Forward: CCATTCCTAAAACCCAAGCA	This study
Wt allele CRM8 Reverse: AGCTGTGAGAGGAAGGGACA	This study
Cre allele Shh_dCreGFP Forward: GGGACAGCTCACAAGTCCTC	Harfe et al., 2004
Cre allele Shh_dCreGFP Reverse: GGTGCGCTCCTGGACGTA	Harfe et al., 2004
Wt allele Shh_dCreGFP Forward: CAACTCCGATGTGTTCCGTT	Harfe et al., 2004
Wt allele Shh_dCreGFP Reverse: CAAGGATCACCAGAAAACATCTG	Harfe et al., 2004
Cre allele Alx4-CreERT2 Forward: CCGTTTGCCGGTCGTGGGCGGCATGG	Rockwell et al., 2022
Cre allele Alx4-CreERT2 Reverse: CGCGCGGCTCCGACACGGGCACTG	Rockwell et al., 2022
Tomato allele R26_LSL_tdTomato Forward: ACATGGCCGTCATCAAAGAG	Madisen et al., 2010
Tomato allele R26_LSL_tdTomato Reverse: CTTGTACAGCTCGTCCATGC	Madisen et al., 2010
Wt allele R26_LSL_tdTomato Forward: AAGGGAGCTGCAGTGGAGTA	Madisen et al., 2010
Wt allele R26_LSL_tdTomato Reverse: CCGAAAATCTGTGGGAAGTC	Madisen et al., 2010
GFP allele R26_LSL_EGFP Forward: CTTCAGCCGCTACCCCGACCACA	Mao et al., 2001
GFP allele R26_LSL_EGFP Reverse: ATCGCGCTTCTCGTTGGGGTCTTT	Mao et al., 2001
Wt allele R26_LSL_EGFP Forward: TGTTCCAATATGGTAGCCAA	Mao et al., 2001
Wt allele R26_LSL_EGFP Reverse: GTGTATTCCTGGCTATCCTAG	Mao et al., 2001
Gli3-A Forward: AGCTGGTAGCCTTAAAATAAGCCAA	Lopez-Rios et al., 2012
Gli3-B Reverse1: GCCTGAAAGAGGTCATCATCACC	Lopez-Rios et al., 2012
Gli3 SA-R Reverse2 : CGTGTCCTACAACACACACTCCAA	Lopez-Rios et al., 2012
