# Supplementary material for: Single-cell profiling of penta- and tetradactyl mouse limb buds identifies mesenchymal progenitors controlling digit numbers and identities
Source: Nat Commun. 2025 Jan 31;16:1226. doi: 10.1038/s41467-025-56221-1 (PMC11785988; doi:10.1038/s41467-025-56221-1)
Supplement: Supplementary file 3 — Description of Additional Supplementary Files [file 41467_2025_56221_MOESM3_ESM.pdf]

Description of Additional Supplementary Files

File Name: Supplementary Data 1
Description: **Gene enrichments for all limb bud celltype clusters.** These gene enrichments were used to determine cluster identities (Supplementary Fig. 3)

File Name: Supplementary Data 2
Description: **Gene enrichments for all wildtype limb bud mesenchymal clusters.** Signature genes for each cluster are listed in bold on top of the Table. The table lists the fold enrichments of gene expression and percentage of expressing cells in all cluster (p-values adjusted). These data were used to determine the most enriched genes allowing identification of cell/tissue types for all eleven mesenchymal clusters.

File Name: Supplementary Data 3
Description: **Specific gene signatures for all mesenchymal clusters.** Core signature genes used for downstream analysis are indicated in bold. Shown are fold enrichment and percentage of cells in the cluster expressing the signature genes

File Name: Supplementary Data 4
Description: **Cell numbers for all mesenchymal clusters per genotype**. These data were used to generate the bar plots and odds ratio analysis shown in Fig. 4.

File Name: Supplementary Data 5
Description: **All DEG for the Msx1+ progenitor clusters P1-P4 for wildtype versus each mutant genotype.** Table lists the fold changes and percentage of expressing cells in wildtype and mutant limb buds. the DEGs are shown in the Volcano plots in Fig. 4 and Supplementary Figure 5.

File Name: Supplementary Data 6
Description: **Percentage of cells with positive pLMP or dLMP scores in clusters P1-P3.** These percentages correspond to the bar plots shown in Fig. 5e. See Source data for statistical values.

File Name: Supplementary Movie 1
Description: **Light sheet Z-stack movie of the anterior lineage in a wildtype forelimb bud at E12.5:** Light sheet microscopy of a wild type mouse forelimb bud at E12.5 showing the spatial distribution and localization along the z axis of the anterior lineage (green) in d1- d3 (SOX9, grey).

File Name: Supplementary Movie 2
Description: **Light sheet Z-stack movie of the anterior lineage in a E1C5 $^{\Delta/\Delta}$ forelimb bud at E12.5.** Light sheet microscopy of an E1C5 $^{\Delta/\Delta}$ mouse forelimb bud at E12.5 showing the spatial distribution and localization along the z axis of the anterior lineage (green) in d1- d3* (SOX9, grey).

File Name: Supplementary Movie 3
Description: **Light sheet 3D volume of the anterior lineage in a wildtype forelimb bud at E12.5.** Light sheet microscopy of a wild type mouse forelimb bud at E12.5 showing the 3D volume of the anterior lineage (green) in d1-d3 (SOX9, grey).

File Name: Supplementary Movie 4

Description: **Light sheet 3D volume of the anterior lineage in E1C5 $^{\Delta/\Delta}$ forelimb bud at E12.5** Light sheet microscopy of an E1C5 $^{\Delta/\Delta}$ mouse forelimb bud at E12.5 showing the 3D volume of the anterior lineage (green) in d1-d3* (SOX9, grey).
