# Supplementary material for: Single-cell profiling of penta- and tetradactyl mouse limb buds identifies mesenchymal progenitors controlling digit numbers and identities
Source: Nat Commun. 2025 Jan 31;16:1226. doi: 10.1038/s41467-025-56221-1 (PMC11785988; doi:10.1038/s41467-025-56221-1)
Supplement: Supplementary file 14 — Reporting Summary [file 41467_2025_56221_MOESM14_ESM.pdf]

nature portfolio

Corresponding author(s): Aimée Zuniga

Last updated by author(s): Jan 7, 2025

Reporting Summary

Nature Portfolio wishes to improve the reproducibility of the work that we publish. This form provides structure for consistency and transparency in reporting. For further information on Nature Portfolio policies, see our Editorial Policies and the Editorial Policy Checklist.

Statistics

For all statistical analyses, confirm that the following items are present in the figure legend, table legend, main text, or Methods section.

n/a	Confirmed	
☐	☒	The exact sample size (*n*) for each experimental group/condition, given as a discrete number and unit of measurement
☒	☐	A statement on whether measurements were taken from distinct samples or whether the same sample was measured repeatedly
☐	☒	The statistical test(s) used AND whether they are one- or two-sided<br>*Only common tests should be described solely by name; describe more complex techniques in the Methods section.*
☒	☐	A description of all covariates tested
☒	☐	A description of any assumptions or corrections, such as tests of normality and adjustment for multiple comparisons
☐	☒	A full description of the statistical parameters including central tendency (e.g. means) or other basic estimates (e.g. regression coefficient) AND variation (e.g. standard deviation) or associated estimates of uncertainty (e.g. confidence intervals)
☐	☒	For null hypothesis testing, the test statistic (e.g. *F*, *t*, *r*) with confidence intervals, effect sizes, degrees of freedom and *P* value noted<br>*Give P values as exact values whenever suitable.*
☒	☐	For Bayesian analysis, information on the choice of priors and Markov chain Monte Carlo settings
☒	☐	For hierarchical and complex designs, identification of the appropriate level for tests and full reporting of outcomes
☒	☐	Estimates of effect sizes (e.g. Cohen's *d*, Pearson's *r*), indicating how they were calculated

Our web collection on statistics for biologists contains articles on many of the points above.

Software and code

Policy information about availability of computer code

Data collection	No commercial, open source or custom code was used for data collection in this study
Data analysis	Single cell RNA seq analysis was performed with various software, for specific details please check the material and methods section of the manuscript.<br>R: version 4.4.1 (2024-06-14).<br>RStudio: Version: 2024.04.2+764.<br>CellRanger: v.6.0.1.<br>Seurat: v.5.1.0.<br>DoubletFinder: v.2.0.4.<br><br>Code availability: All original code has been deposited at Zenodo: https://zenodo.org/doi/10.5281/zenodo.11243709

For manuscripts utilizing custom algorithms or software that are central to the research but not yet described in published literature, software must be made available to editors and reviewers. We strongly encourage code deposition in a community repository (e.g. GitHub). See the Nature Portfolio guidelines for submitting code & software for further information.

Data

Policy information about availability of data

All manuscripts must include a data availability statement. This statement should provide the following information, where applicable:

- Accession codes, unique identifiers, or web links for publicly available datasets
- A description of any restrictions on data availability
- For clinical datasets or third party data, please ensure that the statement adheres to our policy

Single-cell RNA-seq raw and processed data sets have been deposited in the gene expression omnibus (GEO) database under series GSE267005.

Research involving human participants, their data, or biological material

Policy information about studies with human participants or human data. See also policy information about sex, gender (identity/presentation), and sexual orientation and race, ethnicity and racism.

Reporting on sex and gender	no research involving human participants, their data or biological material was performed.
Reporting on race, ethnicity, or other socially relevant groupings	*Please specify the socially constructed or socially relevant categorization variable(s) used in your manuscript and explain why they were used. Please note that such variables should not be used as proxies for other socially constructed/relevant variables (for example, race or ethnicity should not be used as a proxy for socioeconomic status).*<br>*Provide clear definitions of the relevant terms used, how they were provided (by the participants/respondents, the researchers, or third parties), and the method(s) used to classify people into the different categories (e.g. self-report, census or administrative data, social media data, etc.)*<br>*Please provide details about how you controlled for confounding variables in your analyses.*
Population characteristics	*Describe the covariate-relevant population characteristics of the human research participants (e.g. age, genotypic information, past and current diagnosis and treatment categories). If you filled out the behavioural & social sciences study design questions and have nothing to add here, write "See above."*
Recruitment	*Describe how participants were recruited. Outline any potential self-selection bias or other biases that may be present and how these are likely to impact results.*
Ethics oversight	*Identify the organization(s) that approved the study protocol.*

Note that full information on the approval of the study protocol must also be provided in the manuscript.

Field-specific reporting

Please select the one below that is the best fit for your research. If you are not sure, read the appropriate sections before making your selection.

☒ Life sciences ☐ Behavioural & social sciences ☐ Ecological, evolutionary & environmental sciences

For a reference copy of the document with all sections, see nature.com/documents/nr-reporting-summary-flat.pdf

Life sciences study design

All studies must disclose on these points even when the disclosure is negative.

Sample size	scRNAseq: Cell isolation, RNA preparation and sequencing was performed three times with one pair of limb buds for each genotype. The number of total cells analyzed for each genotype was between 11 and 14k cells (details in the material and methods section of the manuscript). The cell numbers are within the range used by previous limb bud scRNAseq studies and is adequate for the level of cell heterogeneity in developing limb buds (Desanlis et al. J. Dev. Biol. 2020, 8(4), 31; https://doi.org/10.3390/jdb8040031; Rouco et al.. Nat Commun 12, 7235 (2021). https://doi.org/10.1038/s41467-021-27492-1).<br><br>Whole-mount in situ (RNA-FISH and WISH) analysis: in mouse embryos at least 3 samples were analyzed per genotype and developmental stage (the exact numbers are included in the figure legends). Gene expression patterns in embryos are extremely robust and based on the standard in the field and our previous experience (Malkmus et al. 2021, doi:10.1038/s41467-021-25810-1) little to no technical and experimental variability is observed for embryos of the same stage and genotype.<br><br>Skeletal analysis: The skeletal digit phenotypes caused by altered Grem1 expression in this study are 100% penetrant, which means that analyzing minimally three embryos is standard in the field. The numbers are included in the figure legend.<br><br>Lineage tracing analysis: Lineage tracing: All experiments were performed with a minimum of three biological replicates per genotype and developmental stage based on the reproducibility of findings and alignment with previously published data. Specifically, results for anterior lineage tracing were consistent with results by from Rockwell et al. 2022 (doi:10.1002/dvdy.328) Harfe et al. 2004 (doi: 10.1016/j.cell.2004.07.024) respectively, validating the experimental approach.
Data exclusions	scRNA-seq analysis: We excluded cells based on the QC thresholds summarized in Methods section. We also removed cell doublets.

	Lineage tracing analysis: it was only applied in cases where no GFP induction was observed for anterior lineage data, as this indicated a lack of lineage labelling. For the posterior lineage, no data were excluded from the analysis.<br>For all other experiments, no data was excluded from this study
Replication	scRNA-seq: 3 biological replicates for each genotype were generated and sequenced independantly. In addition two genetically different Grem1 mutants were used and by identifying DEGs common to both mutants, we increase the robustness and reproducibility of our findings compared to using a single mutant background alone.<br><br>Lineage tracing analysis: Experiments were replicated using embryos from different females across multiple generations of mice. Each cohort included wildtype and control groups, with sample sizes (n) detailed in the corresponding figure legends.
Randomization	Randomization is not possible, as due to the genetic complexity of the analysis, mouse embryos have to be genotyped prior to analysis. However, random allocation is not necessary due to the inclusion of biological replicates and controls. Furthermore, experiments involving RNA-FISH, whole mount immunostaining and skeletal stains were performed by several authors, and gave the same result, which ensures the reproducibility of the results as embryos for the same genotypes were randomly used by different authors.
Blinding	For all experiments involving the comparative analysis of developmentally age-matched limb buds from genetically altered mouse embryos with often complex genotypes blinding is not possible. Deep sequencing for single-cell RNA-seq was done by a sequencing facility who was not aware what individual samples were.

Reporting for specific materials, systems and methods

We require information from authors about some types of materials, experimental systems and methods used in many studies. Here, indicate whether each material, system or method listed is relevant to your study. If you are not sure if a list item applies to your research, read the appropriate section before selecting a response.

Materials & experimental systems

n/a	Involved in the study
☐	☒ Antibodies
☒	☐ Eukaryotic cell lines
☒	☐ Palaeontology and archaeology
☐	☒ Animals and other organisms
☒	☐ Clinical data
☒	☐ Dual use research of concern
☒	☐ Plants

Methods

n/a	Involved in the study
☒	☐ ChIP-seq
☒	☐ Flow cytometry
☒	☐ MRI-based neuroimaging

Antibodies

Antibodies used	Primary antibodies<br>Anti-Human/Mouse Gremlin (1:100, R&D Systems, Cat# AF956)<br>Anti-Phospho-SMAD1/SMAD5/SMAD9 (1:100, Cell Signaling Technology, Cat# 13820)<br>Anti-Phospho-p44/42 MAPK (Erk1/2) (1:200, Cell Signaling Technology, Cat# 9101)<br>Anti-SOX9 (1:400, Millipore, Cat# AB5535)<br>Anti-SOX9 (1:400, R&D Systems, Cat# AF3075)<br>Anti-GFP (1:400, Bio-Rad, Cat# 4745-1051)<br>Anti-dsRed (1:200, Takara Bio, Cat# 632496)<br><br>Secondary antibodies<br>Alexa Fluor 647 donkey anti-goat (1:250 and 1:1000, Invitrogen, Cat# A-21447)<br>Alexa Fluor 555 donkey anti-goat (1:250, Invitrogen, Cat# A-21432)<br>Alexa Fluor 647 donkey anti-rabbit (1:250, Invitrogen, Cat# A-31573)<br>Alexa Fluor 555 donkey anti-rabbit (1:250 and 1:1000, Invitrogen, Cat# A-31572)<br>Alexa Fluor 488 donkey anti-sheep (1:250, Jackson Immuno Research, Cat# 713-545-147)<br>Alexa Fluor 488 donkey anti-rabbit (1:250, Jackson Immuno Research, Cat# 711-545-152)
Validation	All primary antibodies are established reagents that have been used in other mouse studies. Below are examples of studies used for each primary antibody.<br><br>Anti-Human/Mouse Gremlin<br>https://doi.org/10.18632/oncotarget.27050<br><br>Anti-Phospho-SMAD1/SMAD5/SMAD9 (1:100, Cell Signaling Technology, Cat# 13820)<br>https://doi.org/10.1016/j.ydbio.2020.11.004<br><br>Anti-Phospho-p44/42 MAPK (Erk1/2) (1:200, Cell Signaling Technology, Cat# 9101)<br>https://doi.org/10.1242/dev.00669

Anti-SOX9 (Millipore, Cat# AB5535)
https://doi.org/10.1016/j.devcel.2023.02.013

Anti-SOX9 (R&D Systems, Cat# AF3075)
https://doi.org/10.1016/j.celrep.2020.02.037

Anti-GFP (Bio-Rad, Cat# 4745-1051)
https://doi.org/10.1016/j.celrep.2020.02.037

Anti-dsRed (Takara Bio, Cat# 632496)
https://doi.org/10.1038/s41598-019-41770-5

Animals and other research organisms

Policy information about studies involving animals; ARRIVE guidelines recommended for reporting animal research, and Sex and Gender in Research

Laboratory animals	1. Mouse (Mus Musculus) embryos were collected from pregnant mouse females in the Swiss albino background at embryonic days E9.75 - E14.5.<br>2. Pig (Sus domesticus) embryos were collected from pregnant Large White (LW) sows destined for meat consumption at gestational days E21-E24
Wild animals	No wild animals were used in the study
Reporting on sex	Embryonic limb bud development is not sex-biased. Therefore sex was not considered as a variable and embryos of both sexes were used for analysis.
Field-collected samples	No field-collected samples were used in the study
Ethics oversight	All animal experiments were performed in accordance with national laws and approved by the national/local regulatory and ethic committees/authorities. Switzerland (mouse): Regional Commission on Animal Experimentation and the Cantonal Veterinary Office of Basel (national license 1950) in accordance with Swiss laws and the 3R principles. France (pig): The study was approved by the local ethical committee for animal experimentation (CEEA VdL, Tours, France). All methods were performed in accordance with the European Communities Council Directive 2010/63/EU for animal protection and welfare used for scientific purposes. Animals were slaughtered in accordance with European regulation under Directive 2010/63/EU in an experimental slaughterhouse with approval number FR37-175-1.

Note that full information on the approval of the study protocol must also be provided in the manuscript.

Plants

Seed stocks	*Report on the source of all seed stocks or other plant material used. If applicable, state the seed stock centre and catalogue number. If plant specimens were collected from the field, describe the collection location, date and sampling procedures.*
Novel plant genotypes	*Describe the methods by which all novel plant genotypes were produced. This includes those generated by transgenic approaches, gene editing, chemical/radiation-based mutagenesis and hybridization. For transgenic lines, describe the transformation method, the number of independent lines analyzed and the generation upon which experiments were performed. For gene-edited lines, describe the editor used, the endogenous sequence targeted for editing, the targeting guide RNA sequence (if applicable) and how the editor was applied.*
Authentication	*Describe any authentication procedures for each seed stock used or novel genotype generated. Describe any experiments used to assess the effect of a mutation and, where applicable, how potential secondary effects (e.g. second site T-DNA insertions, mosiacism, off-target gene editing) were examined.*
